# Implantación de un programa de diagnostico y tratamiento precoces del VIH en urgencias de un hospital

**DOI:** 10.23938/ASSN.1114

**Published:** 2025-04-25

**Authors:** Hugo Martínez Faya, Amaia Ibarra Bolt, Jorge Abadía Durán, Ana García-Arellano, Carmen Ezpeleta Baquedano, Carlos Ibero Esparza

**Affiliations:** 1 Servicio Navarro de Salud-Osasunbidea Hospital Universitario de Navarra Servicio de Urgencias Generales Pamplona España; 2 Servicio Navarro de Salud-Osasunbidea Hospital Universitario de Navarra Servicio de Microbiología Clínica Pamplona España; 3 Servicio Navarro de Salud-Osasunbidea Hospital Universitario de Navarra Servicio de Medicina Interna Pamplona España

**Keywords:** VIH, Diagnóstico de Infección por VIH, Seropositividad para VIH, Programas de Detección Diagnóstica, Visitas a Urgencias Hospitalarias, HIV, HIV Testing, HIV Seropositivity, Diagnostic Screening Programs, Emergency Room Visits

## Abstract

**Fundamento:**

Determinar la prevalencia de infección por VIH no diagnosticada en personas que acuden al servicio de urgencias hospitalarias (SUH) con determinadas entidades clínicas y describir las características de las seropositivas para VIH.

**Material y métodos:**

Estudio transversal realizado en el SUH del Hospital Universitario de Navarra (Pamplona, España) durante los primeros 18 meses de implementación de un programa de diagnóstico precoz del VIHen SUH. Se incluyeron personas adultas que acudieron al SUH con herpes zóster, infección de transmisión sexual, síndrome mononucleósico, neumonía adquirida en la comunidad, profilaxis post-exposición o *chemsex*. Se les solicitó una serología de VIH no urgente; en caso de resultado positivo, se les informó y se programó una cita en consulta para iniciar tratamiento.

**Resultados:**

Se incluyeron 252 personas (edad media 37,15 años, 63% varones); el 37% consultaron por neumonía adquirida en la comunidad y el 25% por infección de transmisión sexual, y el 27% disponían de serología previa. La serología VIH fue positiva para nueve personas (3,6%), seis entre 35-55 años y siete hombres, con cifras de CD4 <500 células/µL y con carga viral <3.400 copias/mL; todas aceptaron el tratamiento antirretroviral y fueron atendidas en consulta en ≤6 días.

**Conclusiones:**

La implementación del programa de diagnóstico precoz de VIH en nuestro SUH ha demostrado ser una herramienta eficaz para la detección temprana de la infección por VIH, especialmente en pacientes que no acceden regularmente al sistema de salud. Estos datos permiten valorar la efectividad del protocolo, su rendimiento y su capacidad de mejora.

## Abstract (en)

**Background:**

To determine the prevalence of undiagnosed HIV infection among individuals presenting to a hospital emergency department with specific clinical conditions, and to describe the characteristics of those diagnosed with HIV.

**Material and methods:**

A cross-sectional study was conducted in the emergency department of the University Hospital of Navarra (Pamplona, Spain) over the first 18 months following the implementation of an early HIV diagnosis program. Adults presenting with herpes zoster, sexually transmitted infections, mononucleosis-like syndrome, community-acquired pneumonia, post-exposure prophylaxis, or chemsex were included. Non-urgent HIV serology tests were requested. In the event of a positive result, patients were informed and referred for consultation to initiate treatment.

**Results:**

Two hundred and fifty-two patients were included (mean age: 37.15 years; 63% male); of these, 37% presented with community-acquired pneumonia, 25% with sexually transmitted infections, and 27% had previous HIV serology. Nine individuals (3.6%) tested positive: six were aged 35 to 55, seven were male, with CD4 counts < 500 cells/µL and viral loads <3,400 copies/mL. All accepted antiretroviral treatment and were seen in consultation within ≤6 days.

**Conclusions:**

The early HIV diagnosis program in our emergency department proved effective in identifying HIV infection, particularly among individuals with limited interaction with the healthcare system. These findings support the protocol’s utility, its performance, and potential for further optimization.

## INTRODUCCIÓN

El virus de la inmunodeficiencia humana (VIH) es el agente causante del síndrome de inmunodeficiencia adquirida (SIDA), una enfermedad que ha tenido un impacto significativo en la salud global desde su identificación en 1981, siendo uno de los grandes problemas de salud del siglo XX. Desde que se identificaron en Estados Unidos los primeros casos en hombres jóvenes previamente sanos de infecciones oportunistas y ciertos tipos de cáncer, como el sarcoma de Kaposi, se han destinado enormes recursos económicos y humanos para controlar la enfermedad. Gracias a las terapias antirretrovirales, el VIH ha pasado de ser una enfermedad mortal a una condición crónica manejable. Para dimensionar su impacto, 88,4 millones de personas se han contagiado de VIH, que ha causado la muerte de 42,3 millones de ellas en todo el mundo^1^.

A pesar de los avances científicos y los recursos invertidos, tanto en el ámbito económico como en las políticas sociales tras la alarma social de los años 80 y la reducción de casos en la década de los 90, en los últimos años se ha registrado un preocupante aumento de las infecciones de transmisión sexual (ITS), incluyendo el VIH. Este repunte afecta especialmente a personas jóvenes de entre 16 y 20 años, así como a hombres que tienen sexo con hombres (HSH) y otros colectivos vulnerables^2^.

Actualmente la epidemia causada por el VIH sigue sin estar controlada a nivel mundial y es la causa de, aproximadamente, un millón de muertes anuales^2^. El programa sobre el VIH/SIDA creado por la Organización de las Naciones Unidas (ONUSIDA) planteó hace varios años el objetivo 90-90-90 (90% de pacientes con VIH diagnosticados, 90% de pacientes en tratamiento y 90% de pacientes con carga viral indetectable) para el año 2020^3^. A día de hoy, España habría alcanzado los objetivos planteados por el programa ONUSIDA para las personas en tratamiento antirretroviral y para las personas con carga viral indetectable, pero aún estaría algo lejos de alcanzar el 90% de personas diagnosticadas.

Este último año, el programa ONUSIDA ha querido dar un paso hacia delante y ser más ambicioso, planteando los objetivos 95-95-95 (95% de pacientes diagnosticados, 95% de pacientes en tratamiento y 95% de paciente con carga viral indetectable) para el año 2030^4^.

De acuerdo a los últimos datos aportados por el Instituto de Salud Pública y Laboral de Navarra, en la comunidad foral residen 1.250 personas que presentan infección por VIH^5^. De todas ellas, un 16% desconocería su diagnóstico, lo que supone 200 personas no diagnosticadas. Además, cabe destacar que un 45% de los diagnósticos se realiza de forma tardía^5^.

Si ponemos el foco a nivel nacional, se estima que un 13% de las personas que viven con infección por VIH en España desconocen su diagnóstico^6^. En números absolutos, en 2017 se estimó que 151.387 españoles estaban infectados por el VIH, lo que representa una prevalencia del 0,32%. De todas ellas, un 13% desconocería su estado serológico (20.000 personas). Además, casi la mitad (47,6%) de los nuevos diagnósticos de VIH en España se hacen de forma tardía (cifras de CD4 inferiores a 350 células/(l en la primera determinación tras el diagnóstico)^7^, siendo todavía mayor el porcentaje en los pacientes heterosexuales (57%).

El hecho de que haya un gran porcentaje de pacientes que son diagnosticados de manera tardía ocasiona tres importantes consecuencias. En primer lugar, su coste sanitario está incrementado debido a que la tasa de morbilidad y hospitalizaciones se incrementa de manera significativa^8^. En segundo lugar, implica un aumento de la propagación de las infecciones por VIH, ya que la persona no es consciente del diagnóstico y, por tanto, la infección se transmite de modo inadvertido ocasionando una mayor propagación de la epidemia. Según estimaciones, la tasa de transmisión en este tipo de pacientes es de hasta 3,5 veces más, pudiendo ser responsables de hasta el 50% de las nuevas infecciones^9^. Y, por último, los pacientes diagnosticados de forma tardía tienen un riesgo de fallecer 5 veces mayor que los diagnosticados precozmente y, en caso de que el diagnóstico se realice como consecuencia de una enfermedad definitoria de SIDA, el riesgo de fallecer se incrementa hasta 20 veces en el primer año tras el diagnóstico^10^.

Actualmente la vía sexual sigue siendo el modo de transmisión principal en los nuevos diagnósticos. Aproximadamente en los últimos 20 años, el perfil de los pacientes con VIH ha experimentado un cambio; las personas heterosexuales presentan un mayor porcentaje de diagnóstico tardío, del 58,5% en hombres y del 55,8% en mujeres^11^. Por su parte, los HSH son el grupo que presenta mayor número de nuevos diagnósticos, pero con un porcentaje de diagnóstico tardío menor (40,3%)^12^.

Ante este escenario, es necesario aprovechar todas las situaciones que se presenten para diagnosticar pacientes con infección por VIH no conocida, evitando que se conviertan en posibilidades perdidas. Desde finales de la década de los 80, la prueba del VIH se oferta en España en diferentes centros, tanto sanitarios como comunitarios, a diferentes grupos poblacionales^13^. A pesar de ello, y como se ha comentado anteriormente, las tasas de diagnóstico precoz son bajas; por ello, se están llevando a cabo varias estrategias a nivel mundial para ampliar el porcentaje de diagnóstico precoz.

En 2014 se presentó en nuestro país la *Guía de recomendaciones para el diagnóstico precoz del VIH en el ámbito sanitario*^14^ donde se recomendaba y se promovía la realización de una serología ante determinadas circunstancias en los diferentes servicios sanitarios, entre los que se encontraban los servicios de urgencias hospitalarias (SUH). En 2021 se lanzó una iniciativa correspondiente a un programa diseñado para fomentar la detección precoz del VIH en los SUH a nivel nacional y mejorar el acceso rápido al tratamiento, denominado *Deja Tu Huella*^15^. Impulsado por la Sociedad Española de Medicina de Urgencias y Emergencias (SEMES), en colaboración con Gilead y el Grupo de Infecciones en Urgencias (INFURG-SEMES), desde su lanzamiento ha contado con la participación de más de 160 hospitales y más de 200 profesionales. Hasta junio de 2024, se han realizado 170.256 serologías, identificando 1.997 nuevas infecciones, con una tasa de positividad del 1,17%. Se estima que, gracias a estas detecciones tempranas y al inicio oportuno del tratamiento antirretroviral, se han prevenido entre 3.994 y 7.988 nuevas infecciones. Los resultados han sido tan positivos a nivel nacional que se ha decidido revisar el programa para ampliar la detección en dichos servicios y reafirmar o modificar las estrategias a seguir.

Dentro de este planteamiento de ampliación y cambio del programa se realiza el estudio que presentamos a continuación para evaluar la implementación del programa de diagnóstico precoz de VIH en el SUH de nuestro hospital, analizando los datos recogidos durante los primeros 18 meses de funcionamiento. El objetivo es determinar la prevalencia de infección por VIH no diagnosticada en personas que acuden a urgencias hospitalarias y presentan determinadas entidades clínicas, y describir las características de aquellas personas con diagnóstico positivo. Con estos datos se valorará la efectividad del protocolo multidisciplinar implementado, su rendimiento y su capacidad de mejora, y proporcionará una base con la que comparar las nuevas estrategias propuestas en el programa.

## MATERIAL Y MÉTODOS

Se realizó un estudio descriptivo retrospectivo de carácter observacional en el SUH del Hospital Universitario de Navarra (Pamplona, España). El periodo de estudio comprendió desde marzo de 2023 hasta agosto de 2024, abarcando los primeros 18 meses de implementación del programa *Deja Tu Huella*^15^.

Se incluyeron todos los pacientes que acudieron al SUH durante el periodo de estudio, que aceptaron realizarse una serología de VIH no urgente y que cumplían con al menos uno de los siguientes criterios de inclusión, basados en las entidades clínicas indicadoras de posible infección por VIH no diagnosticada:

1. Herpes Zoster: afectación cutánea de un dermatoma como consecuencia de la reactivación del virus Varicela-Zoster (VVZ)^16^, cuya prevalencia es 15 veces mayor en pacientes con infección por VIH^17^, en los cuales produce mayor sintomatología y duración.
2. Infecciones de transmisión sexual (ITS)**:** suponen un problema sanitario a nivel mundial tanto por su incidencia como por la morbilidad que ocasionan^11^; en nuestro país han experimentado un importante aumento, especialmente la sífilis y la infección gonocócica^18^. El VIH y las ITS comparten prácticas de riesgo y mecanismos de transmisión, en este caso la vía sexual, y la realización de un diagnóstico precoz es fundamental para detener su transmisión. Según las recomendaciones para el diagnóstico precoz del VIH del Ministerio de Sanidad, las ITS son una de las enfermedades indicadoras de VIH asociadas a una prevalencia de infección VIH no diagnosticada superior al 0,1%^19^.
3. Profilaxis postexposición (PPE): Hace referencia a aquella profilaxis que se realiza en las primeras 72 horas tras haber presentado una exposición (sexual o laboral) a una fuente cuyo estado serológico es desconocido o positivo en relación al VIH^20^. Actualmente la PPE está protocolizada en todos los SUH del territorio español.
4. Síndrome mononucleósido (SM): Incluye no solo la mononucleosis infecciosa (>90% casos) sino también los denominados síndromes mononucleósidos-*like* (fiebre, adenopatías, odinofagia y exantemas cutáneos), y es casi indistinguible del síndrome retroviral agudo. Según datos del estudio HIDES II, la prevalencia del VIH en pacientes que debutan con SM es del 4,8%^21^.
5. Neumonía adquirida en la comunidad (NAC): En España, 90.000-700.000 personas son diagnosticadas de NAC cada año, un 75% de los casos en los SUH^22^. Actualmente, la NAC es una de las condiciones indicadoras más asociadas a la infección por VIH en nuestro país: en personas seropositivas la incidencia aumenta de 3 a 10 veces en comparación con la población general^23^.
6. Práctica del *chemsex*: uso intencionado de drogas estimulantes para mantener relaciones sexuales por un periodo largo de tiempo (desde horas hasta varios días)^24^. Es una conducta de riesgo, por el consumo de tóxicos y por prácticas sexuales de alto riesgo en pacientes con elevada frecuencia de ITS^25^, en pacientes con baja probabilidad de acudir a atención primaria para la realización de una serología de VIH debido a la falta de anonimato.

El programa estableció un rango de edad para pacientes con HZ o NAC; excluyendo a personas menores de 18 años y mayores de 65 años (en relación con la NAC y el HZ). Esta restricción busca optimizar la identificación de casos en grupos de población con mayor riesgo de infección y asegurar la pertinencia de las pruebas dentro del protocolo de detección.

Para desarrollar el programa, e intentando que la detección fuese lo más completa posible, se estableció un protocolo multidisciplinar que involucraba a los servicios de Urgencias, Microbiología y Enfermedades Infecciosas, así como a Atención Primaria. El protocolo incluía dos pasos: 1) Solicitud de serología de VIH de forma no urgente a todas las personas que acudían al SUH y cumplían, al menos, uno de los criterios de inclusión previamente enumerados. Se solicitaba un consentimiento informado de forma verbal, registrándolo en la Historia Clínica Informatizada (HCI) del paciente y se proporcionaba un documento informativo al paciente sobre la prueba del VIH (Anexo I); 2) Comunicación de resultados. En caso de resultado positivo, el servicio de Microbiología se ponía en contacto con la persona para informarle del resultado, solicitar pruebas adicionales y programar una cita en la consulta de Enfermedades Infecciosas a fin de iniciar el tratamiento antirretroviral lo antes posible. Los resultados negativos se comunicaban a través del personal médico de Atención Primaria, para fomentar la participación activa del paciente en la gestión de su propia salud, y evaluar la necesidad de repetir la prueba con posterioridad.

Se registraron variables demográficas como la edad (años, categorizada en 18-24, 25-34, 35-44, 45-54, 55-64 y >65) y el sexo (hombre/mujer), y variables clínicas como la entidad clínica diagnosticada por la que se le incluyó en el programa, la existencia de serología previa (sí/no), y el resultado de la serología del VIH (positiva/negativa). En caso de resultado positivo, se registró la carga viral (copias/mL), la cifra de linfocitos CD4 (células/(l), si acepta recibir tratamiento o no, y el tiempo de espera tras el resultado positivo hasta la primera valoración médica en la consulta de enfermedades infecciosas e inicio del tratamiento antirretroviral.

El análisis de los datos se realizó con el programa estadístico SPSS versión 29.0.2. Las variables cualitativas se describieron con frecuencias y porcentajes, y las cuantitativas mediante la media y la desviación estándar (DE). La comparación entre variables se realizó mediante Chi-cuadrado (χ^2^) y t de Student, según fueran cualitativas o cuantitativas. Se consideró significativo el valor de p<0,05.

## RESULTADOS

Durante los 18 primeros meses de implantación del programa se incluyeron 252 registros de pacientes que cumplieron alguna de las entidades clínicas especificadas previamente.

La media de edad fue 37,15 años (36,67 para hombres y 37,96 para mujeres), siendo los grupos de 25-34 y 35-44 años los más abundantes; se incluyeron pocos pacientes de 55 años o más. La mayoría eran hombres (63,1%). La entidad clínica de inclusión más frecuente fue la NAC (37%), seguida por ITS y SM; HZ y PPE fueron las menos frecuentes, y no se detectó ningún caso de *chemsex* (Tabla 1).

**Tabla 1:** Frecuencia de sexo, grupos de edad, entidades clínicas y serologías previas en personas incluidas en el estudio [n (%)]

Variables	Global	Hombre	Mujer	p (χ^2^)
	n=252	n=159 (63,1%)	n=93 (36,9%)	
Edad*	37,15 (13,75)	36,67 (14,55)	37,96 (12,31)	0,889
18-24	50 (19,8%)	37 (14,7%)	13 (5,15%)	
25-34	71 (28,2%)	43 (17,1%)	28 (11,1%)	
35-44	58 (23,0%)	36 (14,3%)	22 (8,7%)	
45-54	44 (17,5%)	22 (8,7%)	22 (8,7%)	
55-64	24 (9,5%)	17 (6,7%)	7 (2,8%)	
>65	5 (2%)	4 (1,6%)	1 (0,4%)	
*Entidad clínica*				*0,058*
NAC	93 (36,9%)	61 (24,2%)	32 (12,7%)	
HZ	26 (10,3%)	15 (6%)	11 (4,4%)	
ITS	64 (25,4%)	48 (19%)	16 (6,3%)	
SM	40 (23,8%)	21 (8,3%)	19 (7,5%)	
PPE	29 (11,5%)	14 (5,6%)	15 (6%)	
Chemsex	0 (0%)	0 (0%)	0 (0%)	-
*Serología previa*	68 (27,0%)	45 (28,3%)	13 (14,0%)	*0,009*

*: media (desviación estándar) comparadas con t de Student.

Solo 68 personas (27%) tenían realizada una serología previa, siendo destacable que su frecuencia en hombres duplicó la de en mujeres (28,3 frente a 13,98%, p=0,009). El 67,6% de serologías previas se habían realizado en personas incluidas por NAC (n=31; 45,6%) o ITS (n=15; 22,1%); las 22 serologías restantes se realizaron en personas incluidas por HZ (n=9; 13,2%), SM (n=7; 10,3%) o PPE (n=6; 8,8%).

Nueve de las 252 serologías realizadas en el SUH fueron positivas (3,57%). Seis de ellas correspondían a personas en el grupo de edad de 35-55 años, y otras dos al de 25-34 años; solo dos correspondieron a mujeres (22,2%). Las nueve personas diagnosticadas de infección por VIH presentaron cifras de CD4 inferiores a 500 células/µL, con carga viral inferior a 3.400 copias/mL (Tabla 2). Estos nueve pacientes fueron valorados en consulta de Enfermedades Infecciosas en un plazo máximo de 6 días y todos ellos aceptaron iniciar el tratamiento antirretroviral. Las entidades clínicas por las que se solicitó serología diagnóstica a estos pacientes fueron cinco ITS (55,5%), dos NAC (22,2%), un PPE (11,1%) y un SM (11,1%) (Tabla 2).

**Tabla 2:** Características de las personas con serología positiva para VIH

Variables	Paciente									Global
	1	2	3	4	5	6	7	8	9	
Edad (años)*	35	31	54	36	19	41	35	24	54	36,56 (8,74)
Sexo	H	H	M	H	H	M	H	H	H	7 H (77,8%)
Entidad clinica	ITS	ITS	NAC	PPE	ITS	NAC	ITS	ITS	SM	-
Serologia previa	No	No	No	Sí	No	No	No	No	Sí	2 (22,2%)
CD4 (células/mL)*	490	470	440	478	375	397	323	280	303	395 (79,71)
Carga viral (copias/μL)*	1.250	3.400	2.589	3.100	2.967	2.786	3.104	2.422	2.970	2.732 (627,26)
Aceptación del tratamiento	Sí	Sí	Sí	Sí	Sí	Sí	Sí	Sí	Sí	9 (100%)
Tiempo (días) hasta consulta de Infecciosas*	5	5	4	6	3	4	6	5	4	4,67 (0,81)

*: variables cuantitativas, cuyo valor global se da como media (desviación estándar); H: hombre; M: mujer; ITS: infecciones de transmisión sexual; NAC: neumonía adquirida en la comunidad; PPE: profilaxis postexposición; SM: síndrome mononucleósido.

## DISCUSIÓN

Los resultados de este estudio demuestran el impacto positivo de la implementación de un programa de diagnóstico precoz del VIH en un SUH. La alta tasa de aceptación del cribado por parte de los pacientes y la detección de casos de infección por VIH no diagnosticada, con una prevalencia superior a la nacional (3,57% frente a 0,6-0,9%)^12^^,^^26^, resaltan la importancia de este tipo de iniciativas.

La colaboración multidisciplinar entre Atención Primaria y los servicios de Urgencias, Microbiología y Enfermedades Infecciosas fue fundamental para el éxito del programa. Esta colaboración y compromiso no solo permiten la detección de nuevos casos, sino que en el diagnóstico no fuese tardío en dos tercios de los mismos, ya que mostraban cifras de CD4 superiores a 350 células/μL en esa primera determinación tras el diagnóstico^7^. Además, también garantizan el inicio temprano del tratamiento, lo que mejora el pronóstico de los pacientes^6^ y reduce la transmisión del virus^9^. Además, incluye la implementación de medidas preventivas para evitar nuevos contagios y la realización de un estudio epidemiológico posterior.

En una primera fase esta colaboración permitió una adecuada identificación de los pacientes candidatos al cribado, una rápida comunicación de los resultados y un acceso oportuno al tratamiento para aquellos que fueron diagnosticados, mediante serología no urgente, de infección por VIH.

Cabe mencionar que un 55% de los casos positivos se identificaron en pacientes que acudieron al SUH por una ITS. Este hallazgo concuerda con la tendencia actual de la epidemia de VIH, donde la transmisión sexual juega un papel fundamental^19^. Basándonos en estos resultados podemos decir que el programa ha permitido diagnosticar a pacientes que no hubiesen entrado en un cribado convencional (tras mantener relaciones de riesgo sin protección o tras pinchazo accidental). Esta idea se ha confirmado en las entrevistas mantenidas con esas personas, ya que muy pocas asociaban algunas enfermedades, principalmente el HZ, la NAC y el SM, con la posibilidad de tener una inmunodeficiencia.

Aunque el programa ha demostrado ser efectivo, se identificaron algunas limitaciones que deben ser abordadas en futuras revisiones y actualizaciones. La principal limitación radica en la necesidad de mejorar la adherencia al protocolo por parte del personal medico del SUH para garantizar que todos los pacientes que puedan beneficiarse del protocolo sean correctamente identificados. Debido a la carga de trabajo o a olvidos del personal facultativo, no se solicitó la serología no urgente de VIH a un número nada desdeñable de pacientes, quienes quedaron sin diagnóstico. La implementación de un sistema de recordatorio electrónico en la historia clínica informatizada, que se active al registrar un diagnóstico incluido en los criterios de inclusión, podría mejorar la adherencia al protocolo y reducir las oportunidades perdidas de solicitud de serología y de diagnóstico. Otra de las limitaciones es la formación a todos los profesionales implicados en la asistencia sanitaria: Creemos que una implicación de los profesionales de enfermería en todos los niveles asistenciales (Atención Primaria, Urgencias, etc.) contribuiría a mejorar la implementación del programa.

En comparación con otros estudios realizados en España, la prevalencia de VIH no diagnosticado en nuestro estudio (3,6%) fue superior a la reportada en otros SUH, que oscila entre el 0,6% y el 0,9%^12^^,^^26^. Esta diferencia podría deberse a la selección específica de pacientes con entidades clínicas indicadoras y a la población atendida en nuestro hospital. Es importante destacar que, a pesar de la mayor prevalencia observada en nuestro estudio, el número de casos positivos identificados mensualmente fue inferior al informado en otros estudios con mayor volumen de pacientes. Debemos subrayar también que la distribución por sexo de las serologías positivas es bastante parecida a la que presentan otros estudios^27^.

La implementación de un programa de diagnóstico precoz de VIH en el SUH del Hospital Universitario de Navarra ha demostrado ser una estrategia efectiva para la detección temprana de la infección por VIH, especialmente en pacientes que no acceden regularmente al sistema de salud; siendo prueba de ello la adecuada ratio de positividad que han arrojado los resultados. Además, es necesario recalcar que gracias a la implantación de este programa y, basándonos en los 18 meses analizados, se han conseguido evitar 36 infecciones por VIH basándonos en la ratio de infecciones teórica. Debemos añadir que, a partir de las solicitudes de serología no urgente solicitadas por cada facultativo del SUH, el programa se asoció a una alta tasa de aceptación del cribado por su parte. Además, cabe destacar el breve tiempo de espera tras el resultado positivo hasta la primera valoración médica e inicio del tratamiento antirretroviral (entre 3 y 6 días).

Los resultados de este estudio apoyan la necesidad de implementar programas similares en otros SUH, tanto de la comunidad foral como del resto del país, priorizando la colaboración multidisciplinar, la capacitación del personal sanitario y la utilización de sistemas de recordatorio electrónico para garantizar la adherencia al protocolo. La detección temprana del VIH es fundamental para mejorar el pronóstico individual, reducir la transmisión del virus y avanzar hacia el control de la epidemia.

## Figures and Tables

**Figure:**
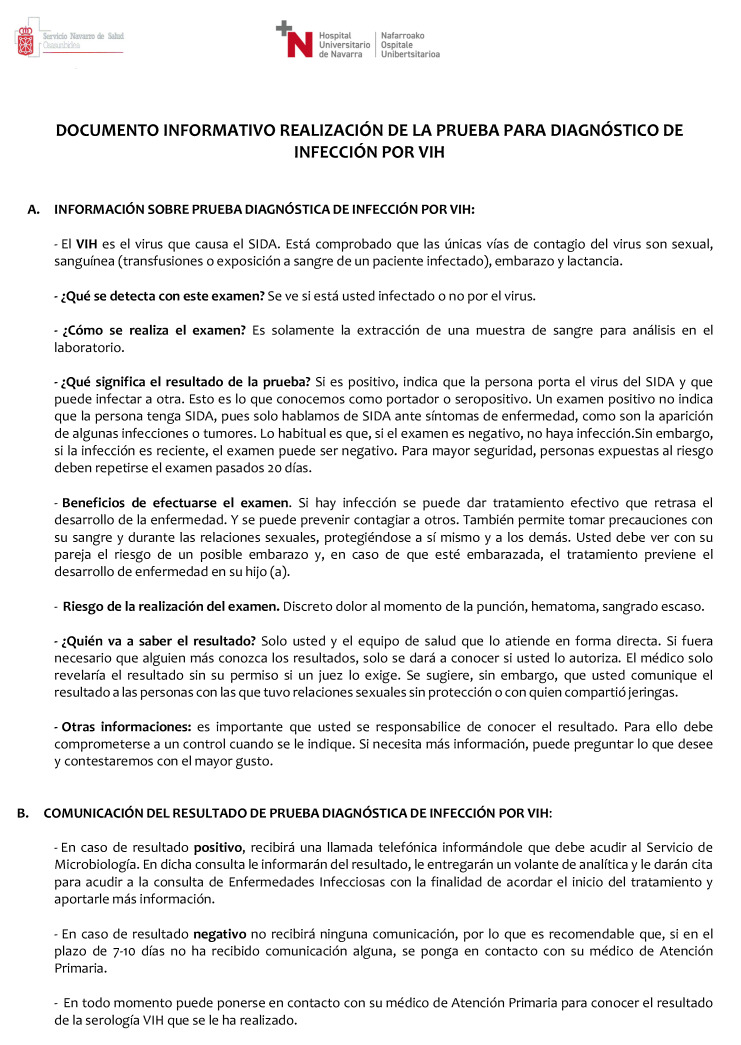
Documento informativo para la realización de prueba diagnóstica de infección por VIH

## Data Availability

Se encuentran disponibles bajo petición al autor de correspondencia.
